# The Intentional Self-Medication of 9/11-Related PTSD Symptoms with Alcohol: 15 Years after the Disaster

**DOI:** 10.3390/ijerph17155327

**Published:** 2020-07-24

**Authors:** Sascha K. Garrey, Alice E. Welch, Melanie H. Jacobson, Robert M. Brackbill, Lisa M. Gargano

**Affiliations:** Department of Health and Mental Hygiene, World Trade Center Health Registry, 30–30 47th Ave, 4th floor, Long Island City, NY 11101, USA; awelch1@health.nyc.gov (A.E.W.); melanie.jacobson2@nyulangone.org (M.H.J.); rbrackbi@health.nyc.gov (R.M.B.); lisa.gargano@gmail.com (L.M.G.)

**Keywords:** alcohol use disorder, self-medication hypothesis, PTSD, September 11th, disaster epidemiology

## Abstract

The self-medication hypothesis may explain the co-morbidity of affective and substance use disorders. Research shows increased prevalence, frequency, and intensity of binge drinking and post-traumatic stress disorder (PTSD) among those directly exposed to the 9/11 terrorist attacks on the World Trade Center (WTC), however, little is known about PTSD symptomology and intentional self-medication with alcohol (ISMA) among this group. We used WTC Health Registry data (*N* = 28,935) to describe the relationship between ISMA and specific symptom clusters of probable 9/11-related PTSD, the number of PTSD symptom clusters endorsed, and binge drinking intensity. Multivariable logistic regression models were used to estimate the adjusted odds ratios (AORs) and 95% confidence intervals (CI). ISMA was most strongly associated with the hyperarousal PTSD symptom cluster (*AOR* = 2.04 [1.88, 2.21]) and the endorsement of one (*AOR* = 1.80 CI [1.65, 1.95]), two (*AOR* = 2.51 CI [2.28, 2.77]), or three (*AOR* = 2.84 CI [2.55, 3.17]) PTSD symptom clusters, indicating a clear dose–response relationship. A significant number of 9/11-exposed persons continue to experience PTSD symptoms and engage in ISMA as a potential coping mechanism. Repeated screenings for self-medicative alcohol use among survivors of mass traumas with PTSD symptoms is of public health importance.

## 1. Introduction

Post-traumatic stress disorder (PTSD) was the most common mental health sequela of the September 11th, 2001, terrorist attacks on the World Trade Center (WTC) in New York City [1]. The disaster exposed thousands of people, including volunteer and professional rescue/recovery workers, lower Manhattan residents, area workers, and passers-by, to a myriad of physical and psychological hazards of varying intensity and duration on the day of 9/11 and in the ensuing weeks and months. Exposure to 9/11 had a significant long-term impact on the physical and mental health among directly exposed populations. Research shows that it resulted in an elevated PTSD prevalence, depending on the population, of 3.8% to 29.6%, immediately after the event and in the years since [2].

Increased alcohol consumption is a short-term effect that has been frequently observed in populations exposed to man-made disasters [3], natural disasters [4] and terrorist attacks [5,6]. For instance, Keyes et al. [7] found that alcohol consumption increased in population samples directly exposed to 9/11 in the months after the event, and over the longer term for those with higher exposure levels. Another study also demonstrated an increased prevalence, frequency, and intensity of binge drinking among those directly exposed to the 9/11 attacks [8] and the among survivors of Hurricanes Katrina and Rita [9]. Comorbidity of PTSD and increased alcohol use is well documented in trauma-exposed populations is [10,11,12,13,14,15,16]. For example, a meta-analysis of substance use and misuse following mass traumas found that, among disaster-exposed individuals, post-disaster drinking was two to six times more likely for those with symptoms of PTSD, compared to those without PTSD symptoms [6].

There are several reasons proposed as explanations for the well documented association between psychological distress and substance abuse. One of the most widely supported theories that describes the increased comorbidity of depression and anxiety-related disorders, such as PTSD, and substance use is the self-medication hypothesis. This theory posits that individuals who experience affect dysregulation or mood-disorders use substances, such as alcohol, to alleviate or cope with their resulting symptoms. In relation to PTSD, the self-medication hypothesis has typically been studied in the context of specific clinical populations [17] or amongst veterans [18]. However, the literature on the self-medication of PTSD symptoms amongst civilian populations exposed to mass-traumas is still nascent. A study assessing the psychiatric impact on survivors of the Oklahoma City bombing found that those who had PTSD and comorbid mental health conditions were the only individuals in the study to use medication or alcohol as a coping mechanism [19]. However, the time frame of this study’s findings are limited to six months after the disaster, thus the associations of delayed cases of PTSD and other longer-term mental health effects with self-medication could not be considered. Another study using data from the National Epidemiologic Survey on Alcohol and Related Conditions (*NESARC-II*) found that close to 20% of individuals with PTSD used substances to relieve their PTSD symptoms [20,21]. However, the PTSD and the self-medication measure used in this study were not tied to a specific traumatic event, thus the authors could not comment on whether there was a common link between the respondents’ reported self-medication behavior and PTSD symptoms. Furthermore, research that examines the self-medication hypothesis in non-institutionalized, civilian populations is limited to relatively small analytical sample sizes and little is known about the intentional self-medication with alcohol (ISMA) of PTSD symptoms and symptom clusters that resulted from 9/11.

Findings pertaining to the relationship of alcohol use and specific PTSD symptomology are also mixed. One study observed that in a sample of civilians seeking treatment for substance use disorders, the frequency of avoidance-cluster PTSD were elevated significantly for individuals with alcohol use disorders [22]. However, another study performed on a similar sample observed that alcohol dependence was most highly correlated with the hyperarousal PTSD symptom cluster [23].

To address these gaps in the self-medication and PTSD literature, data from the World Trade Center Health Registry (WTCHR), a longitudinal cohort study of 71,427 individuals, were used to examine the factors associated with ISMA among the persons directly exposed to the 9/11 attacks who exhibited 9/11-related PTSD symptomology. The use of this cohort provided a unique opportunity to study the self-medication hypothesis within a substantial civilian population sample with an elevated prevalence of PTSD symptomology compared to the general U.S. population.

The goals for the present study were to look more granularly at the association between ISMA and PTSD by (1) examining the association of ISMA with the type of PTSD symptomology endorsed, (2) examining the association of ISMA with the number of concurrent PTSD symptom clusters endorsed, and (3) approximating the relationship of the intensity of ISMA to PTSD severity by describing the relationship among ISMA, the intensity of binge drinking, and PTSD symptom cluster endorsement.

## 2. Materials and Methods

This study’s sample was derived from the World Trade Center Health Registry (WTCHR), a longitudinal cohort study of individuals directly exposed to the 9/11 disaster in New York City. Enrollees in the cohort belonged to one or more of five study eligibility groups: rescue/recovery workers and volunteers, lower Manhattan residents, area workers, school students/staff, and passers-by. Four major WTCHR surveys have been conducted to date: Wave 1 (W1, 2003–2004; *N* = 71,426), Wave 2 (W2, 2006–2007; *N* = 46,600), Wave 3 (W3, 2011–2012; *N* = 43,133), and Wave 4 (W4, 2015–2016; *N* = 36, 862). All surveys from each wave were disseminated to enrollees via mail (i.e., paper-based surveys) or email (i.e., web-based surveys). All survey waves also underwent extensive cognitive testing prior to their implementation. [24]. Details on WTCHR recruitment, enrollment, and survey administration are described elsewhere [25]. We used the data collected from the most recent survey, W4, for the present cross-sectional analysis. This study’s protocol was approved by the institutional review boards at the New York City Department of Health and Mental Hygiene.

For inclusion in this study, enrollees had to have participated in W4 and be at least 18 years old at W4 (*N* = 36,843). Enrollees with missing information on PTSD symptoms at W4 (*N* = 2718) and those who responded “not at all” to all W4 PTSD items were excluded from the analysis (*N* = 4564), as this sequence of PTSD item responses were followed by instructions to skip the question related to ISMA. Those with missing information on ISMA at W4 were also excluded (*N* = 575). Enrollees who reported having never had any type of alcoholic beverage but reported ISMA at W4 were also excluded (*N* = 51). The final study sample was 28,935.

### 2.1. Exposures

The first exposures of interest were the specific PTSD symptom clusters, defined by the American Psychiatric Association’s Diagnostic and Statistical Manual of Mental Disorders (4th ed.; *DSM-IV*) [26] as re-experiencing, avoidance/numbing, and hyperarousal. In this study, specific PTSD symptom clusters were measured using responses to items on the PTSD Checklist-Stressor Specific Version IV, hereon in referred to as the PCL-S, at W4 (see Appendix A for a copy of the PCL-S used on the W4 survey). The PCL-S is a psychometrically robust, 17-item, self-report instrument used to measure the presence and severity of PTSD symptoms that correspond to *DSM-IV* criteria and asks about symptoms in relation to a “stressful experience” [27]. As per PCL-S convention, on the W4 survey, items in the re-experiencing and avoidance domains were queried specifically to 9/11. There was high internal consistency amongst the 17 items used in the PCL-S in this study (α = 0.93). Enrollees were asked how much each of the 17 items on the PCL-S bothered them over the last 30 days on the following Likert scale: 1 = not at all, 2 = a little bit, 3 = moderately, 4 = quite a bit, or 5 = extremely. Items rated at least “moderately” were considered an endorsement of that item. Using *DSM-IV* criteria, the enrollees were categorized as to whether or not they endorsed any of the PTSD symptom domains: re-experiencing (≥1 of 5 items), avoidance/ numbing (≥3 of 7 items); and hyperarousal (≥2 of 5 items) [28].

The second exposure of interest was the number of 9/11-related PTSD symptom clusters endorsed at W4, referred to as PTSD symptom clusters hereon, were measured by summing the instances of endorsing the specific PTSD symptom clusters on the PCL-S, described in detail above. For instance, the enrollees who only endorsed the hyperarousal cluster were considered to endorse one PTSD symptom cluster, whereas the enrollees who endorsed both hyperarousal *and* avoidance clusters were considered to endorse two PTSD symptoms. Our final measure for analysis was a four-level count variable with scores ranging from 0–3 (0 = zero clusters endorsed, 1 = one cluster endorsed, 2 = two clusters endorsed, and 3 = three clusters endorsed).

### 2.2. Outcome

The primary outcome of interest was the ISMA of probable 9/11-related PTSD symptoms at W4, which, drawing upon Turner et al. [29], we defined as reporting having intentionally used alcohol to cope with 9/11-related PTSD symptoms. A dichotomous variable (yes/no) was used to capture whether enrollees had reported ISMA. The ISMA variable was created using the responses to the following question, which was adapted from the *NESARC-II* survey [21], “*In the last 12 month did you drink alcohol to improve your mood or make yourself feel better (when you experienced any 9/11-related PTSD symptoms)?*”. On the W4 survey, only those who rated at least one PCL-S item as “a little bit,” or above, were asked the ISMA question.

### 2.3. Confounders

Variables previously found to be associated with alcohol use were included as potential confounders: binge drinking behavior, history of an alcohol or drug problem, demographic variables (described below), depressive disorder, social support, social integration, and quality of life [8,30].

Binge drinking behavior. Enrollees were asked, “*Considering all types of alcoholic beverages how many times during the last 30 days did you have 5 (for men)/4 (for women) or more drinks on one occasion?*” Drawing from methods used in the National Survey on Drug Use and Health, a nationally representative survey administered to civilian, non-institutionalized U.S. persons in every state annually by the Substance Abuse and Mental Health Services Administration (SAMHSA), binge drinking at W4 was defined as consuming ≥5 drinks on a single occasion in the past 30 days for men and consuming ≥4 drinks on a single occasion in the past 30 days for women. The frequency of binge drinking was defined as the number of binge drinking episodes in the last 30 days. Enrollees were categorized as either none (zero episodes), low-frequency (1–4 episodes), or high-frequency (five or more episodes) binge drinkers [31].

History of alcohol or drug problem A dichotomous (yes/no) variable was used to represent the enrollees’ history of drug or alcohol problems and was derived from the responses to questions designed specifically for the W4 survey. Enrollees who responded “*yes*” when asked if they had, “*ever been told by a doctor or other health professional that you have problems with your use of alcohol or drugs*” were considered to have had a history of an alcohol or drug problem.

Demographic variables. Demographic variables included sex (male/female), race/ethnicity (white, non-Hispanic; black or African American non-Hispanic; Hispanic, American Indian/Alaska Native, Native Hawaiian or other Pacific Islander, Asian, Other), age at W4 (18–34 years, 35–44 years, 45–64 years, and 65+ years), education at W4 (high school/ General Equivalency Degree (GED) or less; at least some college or technical degree; Bachelor’s Degree; Post Graduate Degree), and survey eligibility group (rescue recover worker vs. all other survey eligibility categories).

Depressive disorder. Depressive symptoms at W4 were assessed using a dichotomous variable derived from enrollee responses to eight items on the Patient Health Questionnaire Version 8 (PHQ-8), a validated self-reported instrument that contains eight of the nine *DSM-IV* depression diagnostic criteria [32]. PHQ-8 items on the W4 survey had a high internal consistency (α = 0.92). Scores of ≥10 were considered to indicate moderate to severe depression (1 = *yes PHQ8* ≥ 10; 2 = *no PH PHQ8 ≥ 10*) [33].

Social support status. Social support status was measured via an abridged version of the Medical Outcomes Study Social Support Survey, which uses five items to assess five different domains of functional social support, or the degree to which interpersonal relationships serve particular functions in one’s daily life [34]. Enrollees were asked to rank four items on different domains of functional social support (0 = none of the time, 1 = a little of the time, 2 = some of the time, 3 = most of the time, and 4 = all of the time). In our analysis, continuous scores for social support status ranged from 0 to 20 [35].

Social integration status. Social integration was measured using Brissete et al.’s [36] social integration index, which assessed an individual’s participation in a range of social relationships/networks. Enrollees were asked to indicate using a yes or no response whether certain social integration factors were experienced in the last 30 days. Reporting one or more close friends at the time of the survey was also considered a means of social integration. A final count variable that ranged from 0–4 represented the number of social integrations sources (0 = none, 1 = once source, 2 = two sources 3 = three sources, and 4 = four or more sources) [37].

Satisfaction with life. The extent to which an individual perceived their life as satisfactory, or an individual’s “satisfaction with life,” was measured using the responses to survey questions derived from the Behavioral Risk Factor Surveillance System (BRFSS) survey, a validated questionnaire developed and disseminated nation-wide by the CDC (Center for Disease Control and Prevention). It asked, “*In general, how satisfied are you with your life*?” [38]. The resulting four-level variable reflected the response options: *1* = very satisfied, 2 = satisfied, 3 = dissatisfied, and 4 = very dissatisfied.

### 2.4. Data Analysis

Chi-square tests for dichotomous variables and bivariate logistic regression for the one continuous variable used in the analysis (i.e., social support) were used to assess their associations with ISMA.

We used multivariable logistic regression to model the relationships between the three PTSD symptom clusters (avoidance, re-experiencing, hyperarousal) and ISMA. Adjusted odds ratios (*AOR*) and 95% confidence intervals (*CI*) were computed with those who did not endorse PTSD symptomology and did not report ISMA as the referent. We also performed multivariable logistic regression to model the relationship between the number of PTSD symptom clusters endorsed and ISMA.

A separate descriptive analysis examined the relationship of ISMA, binge drinking, and the number of PTSD symptom clusters endorsed. Chi-square tests were used to assess the associations between the proportions of enrollees reporting ISMA and the different binge drinking groups (i.e., none, low- and high-frequency) stratified by the number of PTSD clusters endorsed (0–3). All analyses were performed on SAS (SAS Institute, Cary, NC, USA) Enterprise Guide, version 9.4.

## 3. Results

### 3.1. Description of Analytic Sample

The largest proportions of the study sample were men (60.0%), non-Hispanic white (71.1%), aged 45–64 years (61.0%) and reported at least some college education or technical degree (29.4%, Table 1). Rescue/recovery workers comprised 45.4% of the study sample. One fifth of enrollees (20.6%) reported the ISMA of 9/11-related PTSD symptoms at W4. Those excluded from our analysis (*N* = 5568) were enrollees who responded, “not at all,” to all PCL items and those who were missing items on the PCL. Demographically, this excluded group did not differ substantially from our analytical sample (data not shown) except for sex: 65.7% of the excluded sample were women and 34.3% were male.

ISMA was more common among men, those aged 18–34 years and 35–44 years, and among those with higher levels of education (Table 1). Among those who reported ISMA, the mean social support score and the prevalence of life satisfaction was lower. As the number of sources of social integration increased, the prevalence of ISMA decreased (Table 2). Of the enrollees with probable depressive disorder, over one-third reported ISMA and of those who reported having a history of an alcohol and/or drug problem, more than half reported ISMA.

All associations tested at the bivariate level were statistically significant for all variables (*p* < 0.001), except for the survey eligibility group (*p* = 0.578).

### 3.2. PTSD Symptomology and ISMA

Almost a quarter of enrollees (23.7%) endorsed the avoidance symptom cluster, 38.8% endorsed the re-experiencing cluster and 38.8% endorsed the hyperarousal cluster (Table 2). We observed that a greater proportion of enrollees reported ISMA in all instances of symptom cluster endorsement compared to those who did not endorse any clusters. The avoidance/numbing cluster had the highest proportion of enrollees who reported ISMA: 35.7% of enrollees who endorsed the avoidance/numbing cluster reported ISMA, while 27.1% of enrollees who endorsed the re-experiencing cluster and 32.3% of enrollees who endorsed the hyperarousal cluster reported ISMA.

In the adjusted model, those who endorsed hyperarousal had two times greater odds of ISMA (*OR* = 2.04, 95% CI [1.88, 2.21]), compared to those who did not endorse the cluster. Compared to those who did not endorse the specific cluster, individuals who endorsed the avoidance/numbing cluster had 1.4 times greater odds of ISMA (*OR* = 1.37, 95% CI [1.25, 1.50]) and those who endorsed the hyperarousal cluster had only a slightly greater odds of ISMA (*OR* = 1.11, 95% CI [1.03, 1.19]) (Table 3).

### 3.3. Number PTSD Symptom Clusters Endorsed and ISMA

One sixth (16.6%) of enrollees endorsed all three PTSD-symptom clusters at W4, whereas 47.7% endorsed zero clusters, 22.0% endorsed one cluster and 13.7% endorsed two PTSD symptom clusters (Table 2).

In the adjusted model, a clear dose–response relationship was observed. Compared to those who did not endorse any symptom clusters, enrollees who endorsed one symptom cluster had close to two times greater odds of ISMA (*OR* = 1.80, 95% CI [1.65, 1.95]), enrollees who endorsed two symptom clusters had 2.5 greater odds of ISMA (*OR =* 2.51, 95% CI [2.28, 2.77]) and those who endorsed all three PTSD symptom clusters had nearly three times greater odds (95% CI *OR* = 2.84 [2.55, 3.17]) of ISMA (Table 4).

### 3.4. Binge Drinking, PTSD Severity, and ISMA

**Figure 1:**
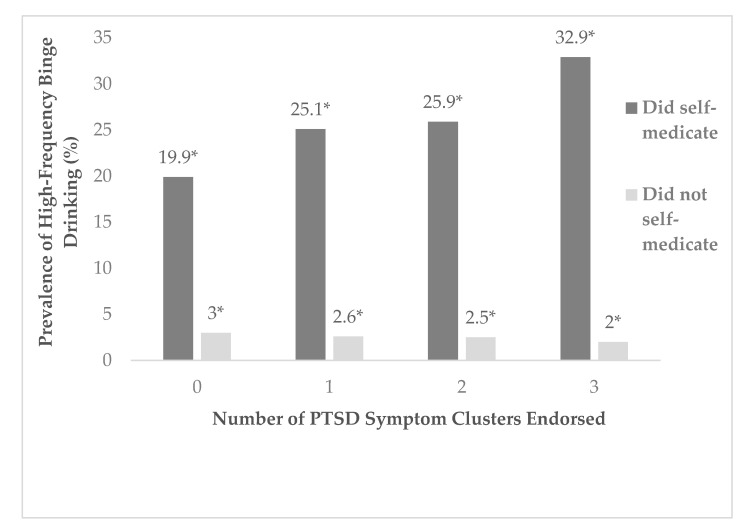
Prevalence of high-frequency binge drinking by the number of post-traumatic stress disorder (PTSD) symptom clusters among (edited out for blind review) who did and did not report intentional self-medication with alcohol, 2016 - 16 (*N* = 28,935); * *p* < 0.001.

The prevalence of high-frequency binge drinking increased as the number of PTSD symptom clusters increased, but only for those who reported ISMA (Figure 1). The prevalence of high-frequency binge drinking amongst those who reported ISMA was lowest amongst enrollees who did not endorse any of the three PTSD symptom clusters (19.9%) and increased with each subsequent level of PTSD cluster endorsement. In this sample, a quarter (25.1%) of those endorsing one PTSD symptom cluster, 25.9% of this sample who endorsed two PTSD symptom clusters, and 32.9% of those endorsing all three PTSD symptom clusters, reported high-frequency binge drinking. The prevalence of high-frequency binge drinking remained relatively constant as PTSD symptom clusters increased for those who did not report ISMA: proportions ranged from 2.0% to 3.0%. All associations in this sub-analysis were statistically significant (*p* < 0.001).

## 4. Discussion

This study examines the topic of ISMA and specific PTSD symptomology and intensity in a post-disaster context among one of the largest disaster-exposed U.S. cohorts with more than 15 years of data. It is one of the few studies to consider the interplay between ISMA, PTSD severity, and PTSD symptomology from a population health perspective [20,29,39]. We observed a strong association between ISMA and the hyperarousal and the avoidance/numbing PTSD symptom clusters, a dose–response relationship between ISMA and 9/11-related PTSD symptom intensity, and a robust association between ISMA and high-frequency binge drinking. Given the complex nature of the interplay between individual PTSD symptoms and alcohol-related behavior, it would be advantageous to continue to apply this line of questioning to larger, more generally representative population samples.

We observed that ISMA was highly associated with specific PTSD symptom clusters, particularly for those who endorsed the hyperarousal and avoidance/numbing clusters. This finding is consistent with PTSD and alcohol-coping literature [20,40,41,42,43]. An important aspect of the self-medication hypothesis is that the substances used by those who self-medicate are not chosen randomly, however, they rather reflect the interplay between the particular psychopharmacological effect of a given substance and the nature of the negative affect for which one seeks relief [44]. Alcohol may be perceived by some to temporarily relieve psychic tension [45,46] and can create the illusion of relief from rigid defenses and feelings of isolation [47]. The PCL-S items, or individual PTSD symptoms, within the avoidance/numbing cluster, such as, “*Feeling distant or cut off from other people*”, and items within the hyperarousal cluster, such as, “*Being super alert or watchful or on guard*”, closely align with the hypothesis that individuals who self-medicate with alcohol do so for the perceived effects to temporarily alleviate feelings of tense emotional states. Our findings are consistent with this aspect of the self-medication hypothesis. Future research may be warranted to consider the relationship of ISMA to the specific PCL-S items within the symptom clusters.

We also observed a positive dose–response relationship between the odds of self-medicating and the number of PTSD symptom clusters endorsed: as the number of PTSD clusters endorsed increased, the likelihood of reporting ISMA increased. Central to the self-medication hypothesis is the assertion that those who experience painful affective states or psychopathology are potentially pre-disposed to the maladaptive use of substances as a means for the temporary relief of related symptoms [44,47]. This was reflected in the present findings in that they demonstrate a clear association between the alcohol use and the severity of a potentially painful affective state: those with the more severe PTSD had a greater prevalence of ISMA. Our interpretation is limited by our self-reported data on potentially sensitive topics, including the severity of PTSD symptoms and alcohol use, which may have led to under-reporting. Because of this, our study may have been subject to social desirability bias, a phenomenon applicable to self-reported data, whereby enrollees may report inaccurately in order to represent themselves in a positive fashion [48]. However, because we were able to measure the number of PTSD symptom clusters endorsed, we were able to expand on the work of Leeies et al. [20], who determined the prevalence for self-medication of PTSD symptoms amongst a general U.S. population sample, but who, given restrictions stemming from their study design, were not able to assess the interplay of self-medication and PTSD severity.

We also found binge drinking and ISMA to be highly associated. When parsed out by the number of PTSD symptom clusters endorsed, the enrollees who reported ISMA consistently had the highest proportions of high-frequency binge drinking compared to those who did not report ISMA, irrespective of the number of PTSD symptom clusters endorsed. This finding supports those of previous studies on binge drinking within similarly exposed populations [8,49,50,51,52]. Given their highly robust association in our sample population, it may be that binge drinking intensity could approximate ISMA intensity. However, this inference is based on an assumption that ISMA is necessarily akin to binge drinking behavior and given that ISMA data was derived from a two-item construct, it is possible that other constructs are driving this association. Future research should aim to expand the ISMA literature to include not only measures of prevalence, but also measures of intensity to further elucidate this association.

This study is subject to several limitations. Firstly, only those enrollees who were aware that at least some of their drinking was in response to distressing mental symptoms resulting from 9/11 reported ISMA. It is likely that there are people in our study sample who self-medicated but did not recognize their drinking behavior as such. Our measure of ISMA does not capture this group. Secondly, due to our cross-sectional study design, we are not able to comment on the longitudinal nature of the association between PTSD and ISMA. The survey used in this study did not include questions regarding drinking behaviors prior to 9/11, therefore we were also not able to distinguish those who engaged in ISMA and/or binge drinking before the exposure to 9/11 from those whose onset of this behavior was post-exposure. All the individuals in the WTCHR database were exposed to 9/11 and we can therefore not make comparisons with an unexposed control group. Furthermore, the ISMA question only appeared on the WTCHR survey as of W4, 15 years after 9/11, so we were not able to gauge the prevalence of ISMA soon after the event nor were we able to consider the individuals who engaged in this behavior at some point after the disaster, but who were no longer engaged with it at W4. Thus, given this study’s design, we are unable to comment with any certainty on the temporal interplay between binge drinking, ISMA, and PTSD. Future research should consider the longitudinal effects of PTSD symptomology on ISMA. Finally, our criterion to include only those with complete PCLs at W4 in the study sample lead to the disproportionate exclusion of women compared to men, which may affect the generalizability of our findings. However, despite these limitations, the sizes of the effects we found were large and statistically significant and indicate that the long-term trajectory of this comorbidity may be an important area for further research.

## 5. Conclusions

Fifteen years post 9/11, a significant number of persons who were exposed to the disaster and continued to experience symptoms of 9/11-related PTSD used alcohol to intentionally self-medicate their symptoms. This dual burden of PTSD and alcohol use related to PTSD demonstrates that, more than a decade later, this population is still in great need of services that address PTSD and alcohol use concurrently, specifically those that intervene on maladaptive coping strategies. Moreover, our findings demonstrate that populations with higher PTSD burdens may be a greater risk for the ISMA of their resulting symptoms. Our findings highlight the importance of repeated screening for self-medicative alcohol use and binge drinking and the inclusion of psychoeducation about substance use in the treatment protocols for the survivors of mass traumas. Findings from this study will inform post-disaster interventions for populations at risk of using alcohol as means to medicate the psychological symptoms of resultant trauma.

## Figures and Tables

**Table 1:** Sociodemographic characteristics of (edited out for blind review) who endorsed at least 1 post-traumatic stress disorder (PTSD) symptom, stratified by intentional self-medication with alcohol, 2015–2016 (*N* = 28,935).

	Intentional Self-Medication with Alcohol						
Variable	Total N	%	Yes, N	%	No, N	%	*p*-Value *
Sex							
Female	11,582	40.0	2233	19.3	9349	80.7	<0.001
Male	17,353	60.0	3728	21.5	13,625	78.5	
Race/Ethnicity							
White, Non-Hispanic	20,568	71.1	4468	21.7	16,100	78.3	<0.001
Black or African American	2598	9.0	470	18.1	2128	81.9	
American Indian/Alaska Native Hawaiian or Other Pacific Islander; Asian; Other	5769	19.9	1,023	17.7	4746	82.3	
Age Group at Wave 4							
18–34 Years	1098	3.8	273	24.9	825	75.1	<0.001
35–44 Years	4064	14.0	1032	25.4	3032	74.6	
45–64 Years	17,651	61.0	3916	22.2	13,735	77.8	
65+ Years	6122	21.2	740	12.1	5382	87.9	
^a^ Education Level at Wave 4							
High School/GED or Less	4367	15.2	780	17.9	3587	82.1	<0.001
At Least Some College or Technical Degree	8458	29.4	1684	19.9	6774	80.1	
Bachelor Degree	8408	29.3	1871	22.3	6537	77.7	
Post Graduate Degree	7459	26.1	1589	21.2	5906	78.8	
Eligibility Group							
RRWs	13,145	45.4	2689	20.5	10,456	79.5	0.578
Other Enrollees	15,790	54.6	3272	20.7	12,518	79.3	

Abbreviations: RRWs: rescue recovery workers; *N*: number; GED: General Equivalency Degree. * *p*-value using chi-square test; ^a^ *N* = 207 missing education.

**Table 2:** Mental health and social characteristics of (edited out for blind review) who endorsed at least 1 PTSD symptom, stratified by intentional self-medication with alcohol, 2015–2016 (*N* = 28,935).

	Intentional Self-Medication with Alcohol						
	Total N	%	Yes, N	%	No, N	%	*p*-Value *
^a^ Number of Sources of Social Integration at Wave 4							
0	217	0.8	54	24.9	163	75.1	<0.001
1	1366	4.8	345	25.3	1021	74.7	
2	12,250	43.4	2905	23.7	9345	76.3	
3	9596	34.0	1883	19.6	7713	80.4	
4	4793	17.0	657	13.7	4136	86.3	
^b^ Social Support Score (0–20)							
Mean	13.9		12.5		14.2		<0.001 **
Standard Deviation	5.3		5.3		5.2		
^c^ Life Satisfaction							
1	6905	24.1	903	13.1	6002	86.9	<0.001
2	16,902	59.0	3441	20.4	13,461	79.6	
3	4068	14.2	1323	32.5	2745	67.5	
4	773	2.7	234	30.3	539	69.7	
Endorse Re-Experiencing Cluster							
Yes	10,947	37.8	2967	27.1	7980	72.9	<0.001
No	17,988	62.2	2994	16.6	14,994	83.4	
Endorse Avoidance Cluster							
Yes	6844	23.7	2441	35.7	4403	64.3	<0.001
No	22,091	76.3	3520	15.9	18,571	84.1	
Endorse Hyperarousal Cluster							
Yes	10,929	37.8	3525	32.3	7404	67.7	<0.001
No	18,006	62.2	2436	13.5	15,570	86.5	
Numbers of Clusters Endorsed							
0	13,790	47.7	1696	12.3	12,094	87.7	<0.001
1	6376	22.0	1340	21.0	5036	79.0	
2	3963	13.7	1182	29.8	27,81	70.2	
3	4806	16.6	1743	36.3	3063	63.7	
History of Alcohol/Drug Problem							
Yes	1433	5.0	782	54.6	651	45.4	<0.001
No	27,502	95.0	5179	18.8	22,323	81.2	

^a^*N* = 713 missing social integration; ^b^ *N* = 641 missing social support score; ^c^ *N* = 287 missing life satisfaction. * *p*-value using chi-square test; ** *p*-value using bivariate logistic regression.

**Table 3:** Adjusted odds ratio (AOR) with 95% confidence intervals (CI) between the endorsement of 9/11-related PTSD symptomology and intentional self-medication with alcohol among (edited out for blind review), 2015–2016 ( *N* = 26,535).

Variable	*AOR* [95% CI]
Endorsement of Avoidance PTSD Symptom Cluster	
No	REF
Yes	1.37 [1.24, 1.50] *
Endorsement of Re-Experiencing PTSD Symptom Cluster	
No	REF
Yes	1.12 [1.03, 1.19] **
Endorsement of Hyperarousal PTSD Symptom Cluster	
No	REF
Yes	2.04 [1.88, 2.21] *

Note: model was adjusted for sex, race/ethnicity, age, education, survey eligibility group, social support, social integration, general life satisfaction, depression status, and history of drug or alcohol problem. * *p* < 0.001. ** *p* < 0.01.

**Table 4:** Adjusted odds ratio (AOR) with 95% confidence intervals (CI) between the number of 9/11-related PTSD clusters endorsed and intentional self-medication with alcohol among (edited out for blind review), 2015–2016.

(*N* = 26,535)	
Variable	*AOR* [95% CI]
Endorsed 0 symptom clusters	REF
Endorsed 1 symptom cluster	1.80 [1.65, 1.95] **
Endorsed 2 symptom clusters	2.51 [2.28, 2.77] *
Endorsed 3 symptom clusters	2.84 [2.55, 3.17] *

Note: model was adjusted for sex, race/ethnicity, age, education, survey eligibility group, social support. social integration, general life satisfaction, depression status, and history of drug or alcohol problem. * *p* < 0.001. ** *p* = 0.078.

## Appendix A

**Table A1:** PTSD Checklist, Stressor Specific, (PCL-S) used on the W4 Survey.

In the Last 30 Days, How Much Have You been Bothered by the Following Problems?					
	Not at All	A Little Bit	Moderately	Quite A Bit	Extremely
a. Repeated, disturbing memories, thoughts, or images of the events of 9/11					
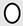
b. Repeated, disturbing dreams of the events of 9/11					
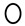
c. Suddenly acting or feeling as if the events of 9/11 were happening again (as if you were reliving it)					
d. Feeling very upset when something reminded you of the events of 9/11					
e. Having physical reactions (e.g., heart pounding, trouble breathing, sweating) when something reminded you of the events of 9/11					
f. Avoiding thinking about or talking about the events of 9/11 or avoiding having feelings related to it					
g. Avoiding activities or situations because they remind you of the events of 9/11					
h. Trouble remembering the important parts of the events of 9/11					
i. Loss if interest in activities that you used to enjoy					
j. Feeling distant or cut off from other people					
k. Feeling emotionally numb or being unable to have loving feelings for those close to you					
l. Feeling as if your future will somehow be cut short					
m. Trouble falling or staying asleep					
n. Feeling irritable or having angry outbursts					
o. Having difficulty concentrating					
p. Being “super alert” or watchful or on guard					
q. Feeling jumpy or easily startled					
